# The Antipsychotic Agent Sertindole Exhibited Antiproliferative Activities by Inhibiting the STAT3 Signaling Pathway in Human Gastric Cancer Cells

**DOI:** 10.7150/jca.34847

**Published:** 2020-01-01

**Authors:** Chunyan Dai, Pei Liu, Xi Wang, Yifei Yin, Weiyang Jin, Li Shen, Yuzong Chen, Zhe Chen, Yiping Wang

**Affiliations:** 1 Digestive Pathophysiology of Zhejiang Province, the First Affiliated Hospital of Zhejiang Chinese Medical University, 54 Youdian Road, Hangzhou, 310006, China; 2 College of Life and Environmental Sciences, Hangzhou Normal University, Hangzhou, 310006, China; 3 Institute of Basic Theory of TCM, China Academy of Chinese Medical Sciences, Beijing 100700, China; 4 Bioinformatics and Drug Design Group, Department of Pharmacy and Center for Computational Science and Engineering, National University of Singapore, 117543, Singapore; 5 Key Laboratory of Integrated Traditional Chinese and Western Medicine for Diagnosis and Treatment of Digestive System Tumor, the First Affiliated Hospital of Zhejiang Chinese Medical University,54 Youdian Road, Hangzhou, 310006, China

**Keywords:** gastric cancer, sertindole, cell apoptosis, cisplatin

## Abstract

Gastric cancer (GC) is the third leading cause of cancer-related death. Although the therapeutic approaches have improved, the 5-year survival rate of GC patients after surgical resection remains low due to the high rates of metastasis and recurrence. Patients with schizophrenia have significantly lower incidences of cancer after long-term drug treatment, suggesting the potential or partially ameliorate the risk of cancer development of antipsychotic drugs. The goal of this study was to explore antipsychotic drugs with an optional effective therapy against gastric cellular carcinoma. We found that sertindole, an atypical antipsychotic, exhibited anti-tumor efficacy on human GC cells *in vitro* and* in vivo*. Moreover, sertindole in combination with cisplatin dramatically enhanced apoptosis-induction in GC cells. In addition, the pro-apoptotic effect of sertindole on GC might in part, involved in inhibition of STAT3 activation and downstream signals, including Mcl1, surviving, c-Myc, cyclin D1. Collectively, these results suggested that sertindole could be a potential anticancer reagent and be an attractive therapeutic adjuvant for the treatment of human GC.

## Introduction

Gastric cancer (GC) remains an important cancer worldwide. It is the fifth most frequently diagnosed cancer and the third leading cause of cancer mortality, with over 1,000,000 new cases in 2018 and an estimated 783,000 deaths (equating to 1 in every 12 deaths globally) 1. The epidemiology data indicate that incidence rates are markedly elevated in Eastern Asia, for example, China, Mongolia, Japan and Korea, while the rates in Northern America and Northern Europe are generally low. Currently, the primary means of treatment is systemic chemotherapy, however, either low response rates or high toxicity are led to the disappointing results. Hence, it is critical to develop new drugs and novel strategies of GC therapeutics.

**Figure 1:**
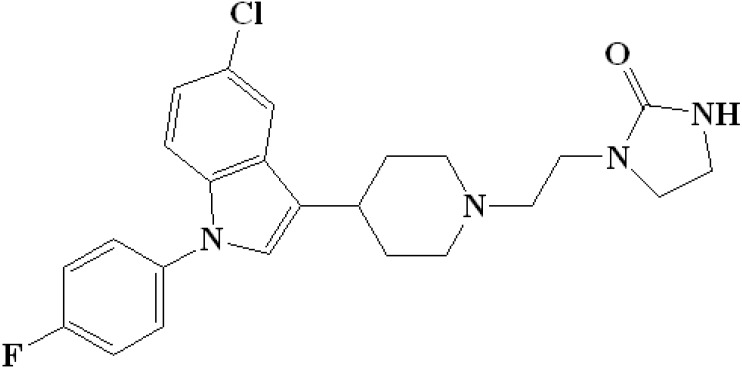
Chemical structure of sertindole.

Recently, epidemiological observations have shown that schizophrenia patients after long-term drug treatment exhibited an overall reduced risk of cancer, which made efforts to reevaluate old drugs with a view to determining their potential as anticancer agents. Some antipsychotic agents like chlorpromazine, thioridazine, trifluoperazine and clozapine have shown anticancer activity 2-6. Sertindole, a phenylindole derivative (Figure 1), is an oral antipsychotic drug available since 1996 for the treatment of schizophrenia in Europe 7. Sertindole is a potent antagonist at dopamine D2, serotonin 5-HT2, and α1-adrenergic receptors with high affinity 8, 9. Sertindole was reported to induce autophagy and autophagy-associated cell death by ROS production in neuroblastoma cells 10. Sertindole also caused breast cancer cell death through autophagy-associated apoptosis via the directly-binding inhibition of 5-HT6 11. However, mechanism of the anticancer effect of sertindole is largely unknown.

Signal transducer and activator of transcription (STAT) proteins are involved in many biological responses and influence cell growth, survival, and metastasis 12-15. Signal transducer and activator of transcription 3 (STAT3), the most strongly associated with tumorigenesis of STAT proteins, can be activated by a variety of upstream signals, including cytokines, growth factors and oncogenes. Generally, the recruited tyrosine kinase JAK can induce STAT3 phosphorylation at tyrosine 705, the phosphorylated STAT3s form self-dimerize and then shuttle to the nuclear for regulating the transcription of its targeting genes 16-17. In contrast to the transient phosphorylation of STAT3 observed under physiological conditions of normal cells, STAT3 often persistently activates in many cancers and in many human cancer models 18. Therefore, targeting inappropriate STAT3 activation is a promising therapeutic strategy. Several strategies have been investigated to target the STAT3 signaling pathway, including design of direct STAT3 small molecular inhibitors, inhibition of upstream tyrosine kinases, and the DNA binding complex 19-21. For example, DU145 cells were observed that cell growth decreased and apoptosis increased when intracellular STAT3 protein levels were reduced by using antisense STAT3 oligonucleotides 22. Akylating agent bendamustine stops the growth of cancer cells by binding to DNA and interfering with its replication through suppression on the SH2 domain of STAT3 23. In the stomach, STAT3 regulates diverse cellular functions including proliferation, basal homeostasis, acute inflammation, angiogenesis and apoptosis 24-25. For instance, STAT3 was reported to constitutively activate in various human GC cells and its inhibition induced apoptosis 26.

In the present study, we investigated the anticancer effect of sertindole in GC. We observed that sertindole could suppress GC cells growth, induce cell apoptosis via suppressing phosphorylation of jak2-stat3 and downstream targets. Oral administration of sertindole suppressed the tumor growth of GC in mice model. To the best of our knowledge, this the first report on the anticancer effects of sertindole targeting jak2-stat3 signaling pathway in GC.

## Materials and methods

### Cell lines

Human gastric cancer (GC) cell lines, including HGC27, MGC803, BGC823 and MNK45 cells (originally purchased from Cell Bank of the Chinese Academy of Science (Shanghai, China)), and the normal human hepatocyte cell line LO2 and human hepatic fetal epithelial cells WRL 68 cells (purchased from Cell Bank of the Chinese Academy of Science (Shanghai, China)) were maintained in RPMI-1640 medium(Gibco®, Hangzhou MultiSciences Biotech Co., Ltd., Hangzhou, China) supplemented with 10% fetal bovine serum (Hyclone). All cells were incubated at 37℃ in a humidified atmosphere containing 5% CO_2_.

### Reagents

Sertindole (empirical formula, C_24_H_26_ClFN_4_O; molecular weight, 440.94) was purchased from Sigma (USA). The sertindole was initially dissolved in dimethyl sulfoxide (DMSO) to obtain a 10 mM stock solution and stored at -20°C, fresh dilutions in medium were made before use. Fetal bovine serum (FBS), 0.25 % trypsin containing EDTA was obtained from Gibco (USA). Cell Counting Kit-8 (CCK-8) was purchased from DoJinDo (Japan).

### *In vitro* cytotoxicity

The *in vitro* cytotoxicity of sertindole was measured by CCK-8, as described in the manufacturer's protocol. Briefly, 5×10^3^ cells per well were plated in 96-well plates and treated with sertindole at various concentrations for 24h. Then, the medium with sertindole was replaced with 200 μL of fresh medium along, 10 μL CCK-8 solution was added into each well and incubated at 37 ºC for 4 h. Absorbance was measured at 450 nm using a spectrophotometer (Bio-Rad, USA).

### Cell apoptosis assays

The Annexin V-FITC Apoptosis Detection Kit (BD Biosciences) was used for apoptosis assays. Cells were seeded in 6-well plates at 2 X 10^5^ per well and harvested after treated with or without sertindole for 24 hours, stained according to the manufacturer's protocol. The cells were analyzed with a FACStar flow cytometer (Canto II), and the data were analyzed using the MODFIT software (BD).

### Protein extraction and western blotting

Total proteins were prepared from cultured cell samples by complete cell lysis (Roche) with protease and phosphatase inhibitors. Denatured proteins (20-50 ug) were separated on SDS-PAGE and transferred to membranes. The following primary antibodies were used: JAK2, p-JAK2, STAT3, pSTAT3 (Tyr 705), Mcl-1, Survivin, c-Myc, cyclin D1, PARP, Caspase 3, Caspase 9, XIAP, bcl 2, GAPDH, β-actin, tublin (all from Cell Signaling Technology). The bands were scanned using ChemiDocXRSt Imaging System (Bio-Rad).

### Tumor xenograft model

Male BALB/c nu/nu nude mice at the age of 4-5 weeks (Zhejiang Academy of Medical Sciences), were housed at a specific pathogen-free environment in the Animal Laboratory Unit, Zhejiang Chinese Medical University, China. Mice were administered with sertindole (10mg/kg or 20mg/kg) or cisplatin (5mg/kg) or equivalent concentration of DMSO every two days. All mice were sacrificed after 16 days. All procedures involving animals were conducted with the approved of the Committee on Animal Care in the First Affiliated Hospital of Zhejiang Chinese Medical University.

### Statistical analysis

All numeric values are shown as mean ± SD. Each set of experiment was repeated at least three times. Statistically significant differences between experimental and untreated control groups were detected using Student's unpaired t-tests. Difference was considered to be significant if p <0.05.

## Results

### Sertindole suppresses proliferation of GC cells

**Figure 2:**
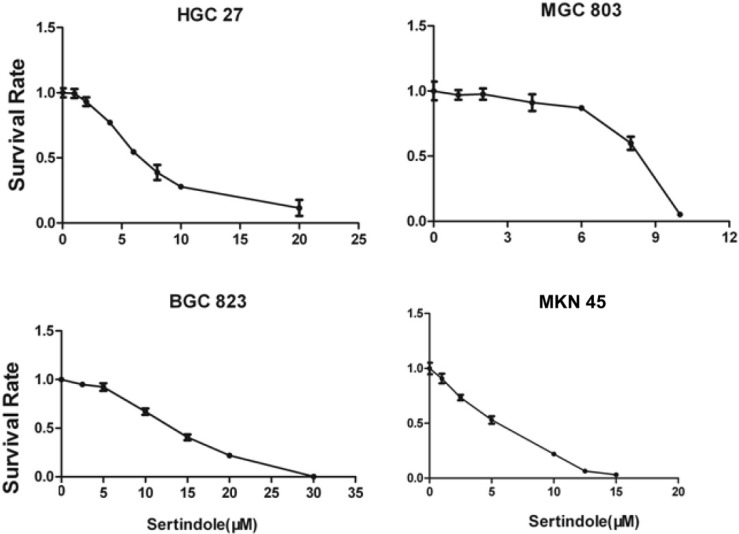
**Sertindole inhibited gastric cancer (GC) cell proliferation *in vitro*.** The growth inhibitory effect of sertindole was measured using the CCK-8 assay. Gastric cancer cell lines including HGC27, MGC803, BGC823 and MKN45 cells were treated with varying concentrations of sertindole (from 0 to 30 μM for 24 h). The experiments were performed in triplicate, and the data are presented as the mean ± standard deviation (SD) of three separate experiments.

To evaluate the growth-suppressive effects of sertindole, we first performed the cytotoxicity assay in HGC27, MGC803, BGC823 and MNK45 cells. The cells were treated with varying concentrations of sertindole for 24 hours. Our results showed that treatment with increasing concentrations of sertindole significantly suppressed the growth of all the four GC cell lines in a concentration-dependent manner. The detailed IC50 values of sertindole against different cell lines were shown as table 1, the IC50 of sertindole after 24 hours treatment ranged from 4 to 16 μM in all the four cell lines (Figure 2). In addition, we measure IC50 of sertindole against normal human hepatocyte cell line LO2 and human hepatic fetal epithelial cells WRL 68 cells. The IC50 values of LO2 and WRL 68 were 10.31±0.17 and 12.71±0.33 μM, respectively. The IC50 curve of these two cell lines was shown as Supplementary figure 1. Compared to gastric cancer cells, the IC50 of the two normal hepatic cell lines was lower than BGC823 (15.85±2.19 μM), but higher than the other three gastric cancer cell lines, suggesting that sertindole couldn't specifically inhibit the growth of gastric cancer cells. Collectively, these results suggested potential cytotoxic effects of sertindole in GC cells.

### Sertindole induced apoptosis of GC cells

**Figure 3:**
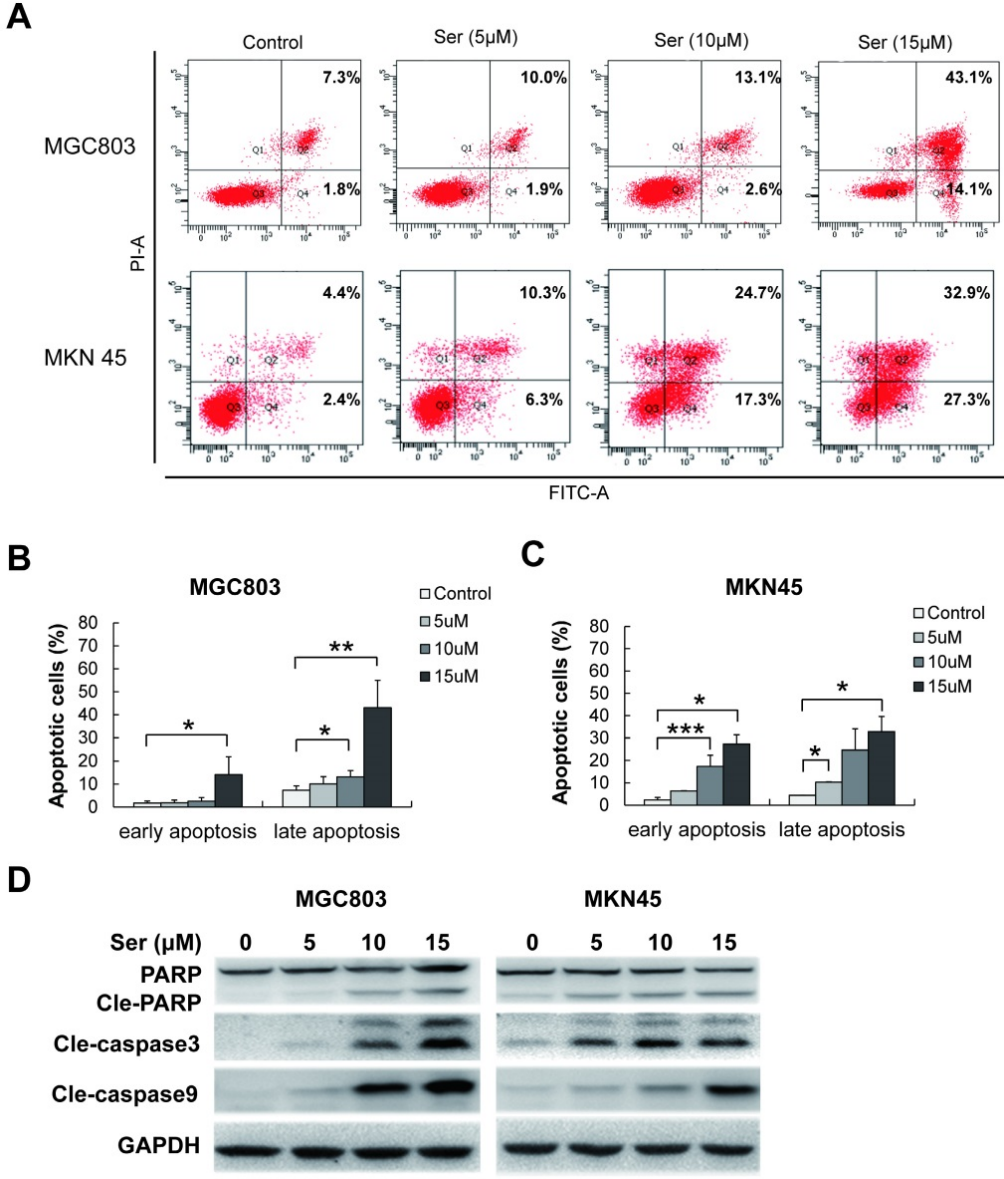
**Sertindole induced cell apoptosis in GC cells. (A)** MGC803 and MKN45 cells were treated with gradient concentrations of sertindole for 24 h. Then, the cells were stained with PI at 37 °C for 30 min and measured by flow cytometry after collection. **(B)** Quantitative analysis of apoptotic cells. The percentage of cells in different phases of cell apoptosis was represented by a bar diagram. Data are presented as the mean ± SD of three independent experiments. **(C)** Effects of sertindole on the expression of apoptotic-associated proteins. **(D)** MGC803 and MKN45 GC cells were treated with 0, 5, 10 and 15 μM of sertindole for 24 h, and cell lysates were subjected to western blot analysis with cleaved PARP, caspase-3 and caspase-9 antibodies.

To determine the features of sertindole-induced GC cell growth inhibition, flow cytometric analysis was carried out in MGC803 and MNK45 GC cells for apoptosis analysis. Annexin V/PI staining was performed after GC cells were treated with different dosages of sertindole. Early and late apoptotic cells were counted. After 24 hours, sertindole-treated MGC803 and MNK45 GC cells exhibited a dose-dependent increase in annexin V-positive cells compared with the control cells (Figure 3A-C). To investigate the apoptotic-associated signal transduction in GC cells after sertindole treatment, MGC803 and MNK45 GC cells treated with sertindole were analyzed with Western blot. As shown in Figure 3D, cleaved PARP, caspase-3 and caspase-9 was increased dose-dependently. These results indicated that sertindole induced apoptosis of GC cells.

### Sertindole inhibited constitutive JAK2-STAT3 phosphorylation in GC cells

**Figure 4:**
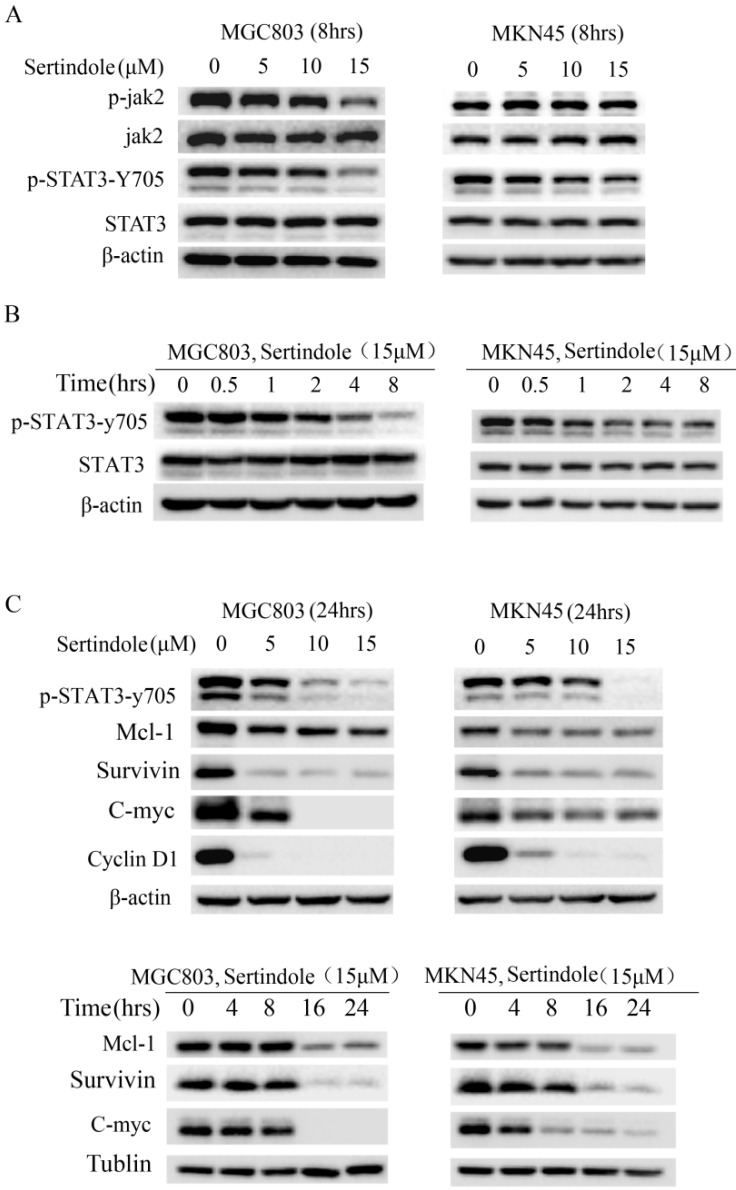
**Sertindole suppressed GC cell proliferation through inhibition of JAK2-STAT3 signal pathway. (A)** The GC cells were treated with sertindole at gradient concentrations for 8 h. The cell lysates were then separated by 12% SDS-PAGE electrophoresis, and the levels of proteins related to JAK-STAT3 signaling pathway were detected. **(B)** MGC803 and MKN45 cells was treated with 15 μM sertindole for 0, 0.5, 1, 2, 4 and 8 h, the levels of STAT3 and phosphorylated STAT3 (p-Stat3-y705) were detected by western blot analysis. **(C)** Western blot determined the levels of STAT3 downstream targets, including Mcl1, surviving, c-Myc, cyclin D1.

To elucidate the molecular mechanism of the growth-suppressive effects of sertindole, we carried out Western blot analysis of whole-cell lysates of MKN45 and MGC803 cells treated with 0, 5, 10 and 15 μmol/L sertindole. As shown in Figure 4A, the treatment of sertindole (8 hrs) significantly inhibited STAT3-try 705 phosphorylation in MKN45 and MGC803 GC cells, and phosphorylation of JAK2 was significantly down-regulated in MGC803 GC cells. The inhibition on JAK2 and STAT3-try 705 phosphorylation was in a concentration-dependent manner. Moreover, we treated MGC803 and MKN45 cells with sertindole at 15 μmol/L for 0, 0.5, 1, 2, 4, 8 hours, the inhibition of STAT3-try 705 phosphorylation was observed in a time-dependent manner (Figure 4B). In addition, the levels of downstream targets of STAT3, including Mcl-1, survivin, c-Myc and cyclin D1, were obviously decreased by sertindole treatment. The inhibition of sertidole on these proteins was time- and dosages-dependent (Figure 4C). Taken together, these results indicated that sertindole suppressed GC cell survival by inhibiting the activation of JAK2-STAT3 signal pathway and expression of downstream targets.

### Sertindole synergistically inhibits GC cells *in vitro* while combined with cisplatin

**Figure 5:**
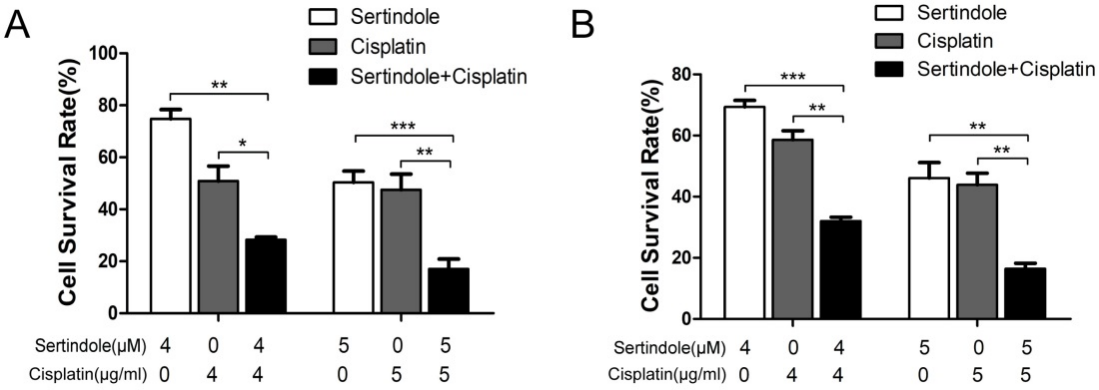
** Combined treatment of sertindole and cisplatin to GC cells reduced cell viability.** The effects of sertindole alone, cisplatin alone or sertindole together with cisplatin on cell viability were measured by cck8 assay. Combined treatment inhibited viability in MGC803 **(A)** and MKN45 **(B)** cell lines in a dose-dependent manner.

To determine whether sertindole could sensitize GC cells toward chemotherapeutic agents, we treated MKN45 and MGC803 cells with sertindole or cisplatin alone, sertindole together with cisplatin for 24 hours, then analysis the percentage of survival cells by cck8 assay. The results showed that higher concemtration of sertindole or cisplatin alone led to lower cell survival rate, and the cell survival rate was significantly decreased after treatment of cells with combination of sertindole and cisplatin (Figure 5).

**Figure 6:**
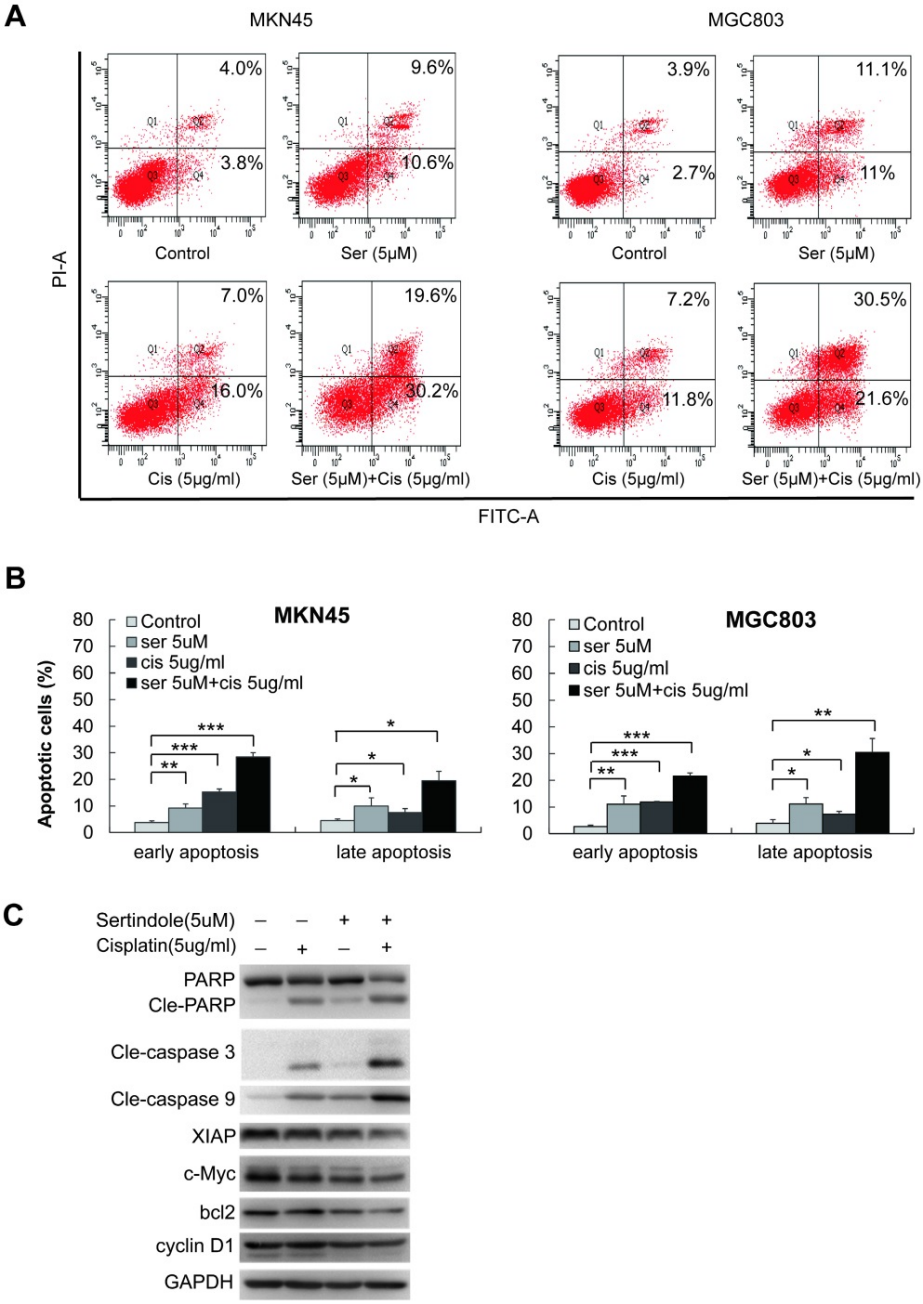
** Sertindole potentiated the apoptotic-induction effects of cisplatin in GC cells. (A)** The MKN45 and MGC803 cells were treated with sertindole alone, cisplatin alone or sertindole in combination with cisplatin for 24 h. The cells were collected and stained with PI at 37 °C for 30 min, then measured by flow cytometry. **(B)** The histogram of cell apoptosis rates. Data are presented as the mean ± SD of three independent experiments. **(C)** The MKN45 cells were treated with sertindole alone, cisplatin alone or sertindole in combination with cisplatin for 24 h. The cell lysates were then separated by 12% SDS-PAGE electrophoresis, and cell apoptotic- and growth-related proteins expressions were detected by western blot analysis.

Next, we examined the combination of sertindole and ciaplatin on apoptotic cells by using flow cytometry. As shown in figure 6A-B, while treating with 5 μg/ml of cisplatin for 24 hours, the early and late apoptotic cells were 10.0% and 8.9%, respectively, in MKN45 cells. The combined treatment of sertindole and cisplatin in MKN45 increased the early and late apoptotic cells to 20.7% and 26.9%, respectively. The similar phenomenon was observed in MGC803 cells, the treatment of sertindole together with cisplatin obviously increased the percentage of apoptosis cells than the addition of sertindole or cisplatin alone, suggesting that the combined sertindole and cisplatin treatment provided a significantly higher cytotoxic effect than cisplatin treatment alone. Next, we examined the changes in molecules related to the cytotoxic effects by Western blot. After adding sertindole together with cisplatin, MKN45 cells showed a higher cleaved PARP, cleaved caspase-3 and cleaved caspase-9 activity as compared with cells with single drug. Moreover, the expression of proteins related to cell growth and anti-apoptosis, such as XIAP, bcl2, c-Myc and cyclinD1 were down-regulated (Figures 6C). Taken together, these results indicated that sertindole enhanced cisplatin-mediated GC cell cytotoxic effects and sensitized GC cells toward ciaplatin.

### Antitumor effects of sertindole in a xenograft GC model

**Figure 7:**
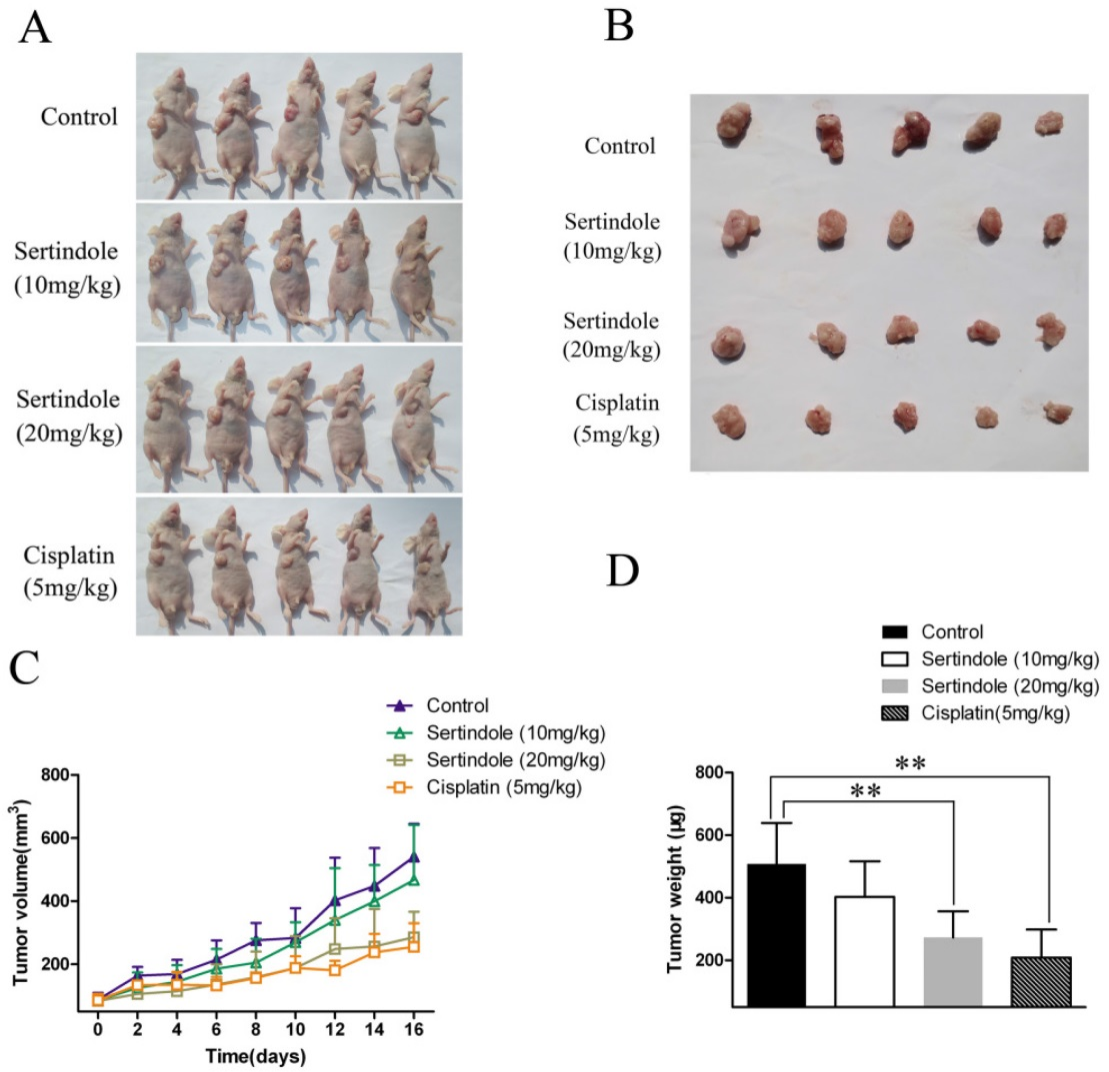
** Sertindole inhibited tumor growth of MGC803 cells in nude mice. (A)** The transplanted tumors in nude mice; **(B)** The transplantation tumors removed from nude mice; **(C)** Tumor volumes were measured on the indicated days; **(D)** The weight of the transplanted tumor. **P<0.01 vs. control.

We then examined the ability of sertindole to reduce the growth of human GC cells subcutaneously implanted in nude mice. MGC803 GC cells were injected subcutaneously into the right flanks of the mice. One week after tumor cell injection, the animals were randomized into 4 treatment groups that tumor volumes were similar among the groups. The four groups of mice were received vehicle, sertindole alone (10 mg/kg/day, oral gavage), sertindole alone (20 mg/kg/day, oral gavage), cisplatin alone (5 mg/kg/day, oral gavage) treatment. Treatment began two weeks after implantation and continued for up to 16 days. Tumor diameters were measured every 2 days. Animals were killed 16 days after the initiation of treatment, and the diameters of the excised tumors were measured. Comparatively, mice that received sertindole alone (20 mg/kg/day, oral gavage) showed significantly lower tumor burden than those that received vehicle or sertindole alone (10 mg/kg/day, oral gavage). Sertindole (20 mg/kg/day, oral gavage) alone-treated mice demonstrated a similar level of tumor burden as the cisplatin (5 mg/kg/day, oral gavage) group (Figure 7). The nude mice treated with 20mg/kg of sertindole had a bright coat, normal appetite and slightly lighter weight than the control group, but the body weight was the same as the cisplatin group (Supplementary figure 2).

## Discussion

Gastric cancer is the third leading cause of cancer-related death. Although the therapeutic approaches have improved, the 5-year survival rate of GC patients after surgical resection remains low due to the high rates of metastasis and recurrence. Moreover, the multidrug resistance is the other important reason for the failure of GC therapy. Recent years, reusing and repurposing of 'old' drugs is increasingly becoming an attractive strategy because of its lower overall development costs and shorter development timelines, as well as the use of de-risked compounds. The goal of this study was to explore old drugs with an optional effective therapy against gastric cellular carcinoma. We found that sertindole, an atypical antipsychotic, exhibited anti-tumor efficacy on human GC cells *in vitro* and* in vivo*. Moreover, sertindole in combination with cisplatin dramatically enhanced apoptosis-induction in GC cells. In addition, the pro-apoptotic effect of sertindole might involve in inhibition on activation of JAK2-STAT3 signaling pathway.

Antipsychotic drugs have been used for decades in various psychiatric clinical settings, epidemiological studies with various patient populations have demonstrated that patients with schizophrenia have significantly lower incidences of cancer than the general population, suggesting the potential or partially ameliorate the risk of cancer development of antipsychotic drugs. Trifluoperazine, an existing phenothiazine-like antipsychotic drug, was repurposed as a potential anti- cancer stem-like cell agent by overcoming epidermal growth factor receptor-tyrosine kinase inhibitor and chemotherapy resistance 3. Penfluridol effectively reduces the growth of primary triple-negative breast cancer tumors and especially metastatic growth in the brain by inhibiting integrin signaling 27. However, the underlying molecular mechanisms are yet to be elucidated. Sertindole is an atypical antipsychotic, which gives a lower incidence of extrapyramidal side effects and favorable metabolic profile at clinically effective doses than typical antipsychotic drugs 28-29. After a reevaluation of its risks to cardiovascular safety and benefits to refractory patients, sertindole is available as a second-line choice for patients intolerant to other antipsychotic agents 30-31. In the present study, we observed suppression effects of sertindole on GC cells growth with an IC_50_ concentration range from 4 to 16 μM, and oral administration at 20mg/kg of sertindole suppressed the tumor growth of GC cells in mice model. Compared the administration of up to 40 mg/kg sertindole in rats, which the dose is 10-fold greater than the maximal therapeutic dose of psychosis and does not elicit extrapyramidal symptom 32, the sensitivity of sertindole to GC cells was much higher. These results indicated that the potential anti-tumor effect of sertindole in GC.

Sertindole, a phenylindole derivative, is a potent and long acting compound as an antipsychotic drug. *In vivo* behavioural experiments showed that sertindole was dose-dependently binds to three receptors, which has the most pronounced effect on 5-HT2 receptors, lower effect on αl-adreno receptors and the lowest effect on striatal D2 receptors on dopamine neurons in the rat 33. Sertindole proved to be significantly better than first-generation drugs on relapse prevention. Moreover, sertindole was better than first-generation drugs refer to improve the life quality in schizophrenic patient 34. A few papers explored the anti-cancer effects of sertindole 8-10, however, the underlying mechanism of the anti-tumour effect of sertindole remains less well characterised. In this study, we found that sertindole induced GC cell apoptosis, inhibited the phosphorylation of JAK2 and STAT3 at tyrosine 705 in time- and dose-dependent. STAT3 is in general transiently activated in normal cells but constitutively activated in a variety of solid tumors, including breast cancer, prostate cancer, head and neck cancer and GC cancers 35-38. STAT3 is a key regulator of convergence of multiple oncogenic signaling pathways and is regarded as a “master regulator” that plays critical roles in human cell cycle, apoptosis, angiogenesis, stemness, metastasis to immune evasion 35. In this study, the activation of STAT3 was suppressed when GC cells were exposed to sterindole, suggesting its therapeutic potential on GC. Further, the STAT3 has long been believed to act as a classic transcription factor to regulate the expression of a large number of target genes. For example, the protein encoded by the proto-oncogene c-Myc is a downstream target of STAT3 that is thought to be involved in tumor initiation and development. Activation of c-Myc is crucial for sustained tumour cell proliferation and survival 39-42, while suppression of c-Myc expression induces tumour regression in different tumour types 43 and promotes rapid tumour deterioration by triggering apoptosis or senescence 44. In the present study, sertindole suppressed the expression of c-Myc in a dose-dependent manner. Taken together, sertindole could suppress the phosphorylation of STAT3 and c-Myc expression level, suggesting that the pro-apoptotic effect of sertindole on GC might in part, involve in inhibition of STAT3-c-Myc activation.

In conclusion, we showed that sertindole inhibited cell proliferation and induced cell apoptosis in GC cells. This event was associated with inhibition in JAK2-STAT3 signaling pathway and downstream signals, including Mcl1, surviving, c-Myc, cyclin D1. Moreover, sertindole exhibited anti-tumor efficacy on a xenograft GC model. In addition, sertindole in combination with cisplatin dramatically enhanced apoptosis-induction in GC cells. Combination therapy with sertindole and cisplatin might be a useful method to treat GC cells. All our data suggested that sertindole might be a promising candidate as an antitumor agent against GC.

## Supplementary Material

Supplementary figures and tables.

## Figures and Tables

**Table 1:** IC_50_ values of sertindole against different human gastric cancer cell lines.

Cell lines	IC_50_(μM)
HGC27	9.75±2.62
MKN45	4.97±1.00
MGC803	6.83±1.85
BGC823	15.85±2.19
