# Supplementary material for: The Antipsychotic Agent Sertindole Exhibited Antiproliferative Activities by Inhibiting the STAT3 Signaling Pathway in Human Gastric Cancer Cells
Source: J Cancer. 2020 Jan 1;11(4):849–57. doi: 10.7150/jca.34847 (PMC6959018; doi:10.7150/jca.34847)
Supplement: Supplementary file 1 — Supplementary figures and tables. [file jcav11p0849s1.pdf]

Supplementary figure 1. Sertindole inhibited normal hepatic cell lines cell proliferation in vitro.

The growth inhibitory effect of sertindole was measured using the CCK-8 assay. Two normal hepatic cell lines LO2 and WRL68 cells were treated with varying concentrations of sertindole (from 0 to 20 μM for 24 h). The experiments were performed in triplicate, and the data are presented as the mean ± standard deviation (SD) of three separate experiments.

Supplementary figure 2. Body weight of nude mice was measured on the indicated days.

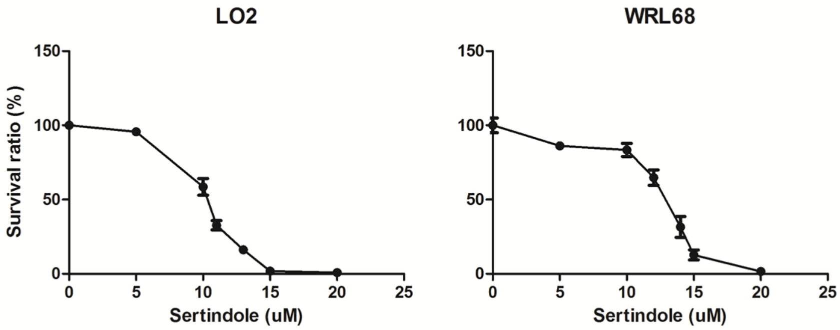

Supplementary figure 1

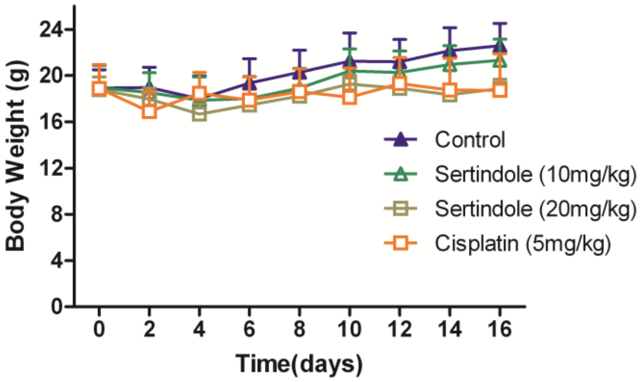

Supplementary figure 2
